# Social support quality and availability affects risk behaviors in offenders

**DOI:** 10.1186/s40352-016-0033-y

**Published:** 2016-02-25

**Authors:** Stephanie A. Spohr, Sumihiro Suzuki, Brittany Marshall, Faye S. Taxman, Scott T. Walters

**Affiliations:** 1 University of North Texas Health Science Center, Fort Worth, USA; 2 George Mason University, Fairfax, VA USA

**Keywords:** Criminal justice, Substance abuse, STD/HIV, Social support

## Abstract

**Background:**

People involved in the justice system are at 2.5 times the risk of HIV infection compared to the general population, which is further complicated by substance abuse. The purpose of this study was to evaluate the role of social network *quality* and *quantity* on unprotected sex, criminal risk, and substance use.

**Methods:**

We used data from 330 drug-involved offenders. Structural equation modeling (SEM) was used to model and test path directionality and magnitude between the latent constructs of social support quality and quantity on risky behaviors.

**Results:**

The SEM indicated the latent construct of social support quality was significantly associated with reduced sexual risk behavior (β = −0.27), criminal risk (β = −0.26), and reduced substance use (β = −0.33). Additionally, the proposed model found that social support quantity was significantly positively associated with increased sexual risk behavior (β = 0.40) and substance use (β = 0.20).

**Conclusions:**

Social support quality is an important predictor of risky behaviors; as the quality of an offender’s social support increases, engagement in risky behaviors decreases. Probationers who had broader social support availability also had increased substance use and unprotected sex. Probation systems may be able to reduce substance use and STD/HIV infection risk in offenders by strengthening the quality of social support networks.

## Background

More than 4.7 million people were under community supervision (probation and parole) in the United States in 2013, and over two million people enter probation every year (Herberman & Bonczar 2014). While rates of offenders under community supervision have been decreasing since 2007, probationers and parolees still account for the largest segment of the criminal justice system--nearly three times that of incarceration populations (Herberman & Bonczar 2014). This high rate of criminal justice involvement creates a significant public health need and calls for the development of services to reduce rates of incarceration and recidivism.

Like many vulnerable populations, offenders experience a variety of health issues at a disproportionate rate as compared to other groups. One such health condition is human immunodeficiency virus (HIV) or acquired immunodeficiency syndrome (AIDS). The rate of AIDS cases among justice-involved persons is 2.5 times higher than the general population (Maruschak & Beavers 2010; Center for Disease Control and Prevention 2012), due in part to increased risk factors and engagement in risky behaviors. Increased rates of HIV in the community are partially attributed to disrupted partnerships and economic instability (Blankenship & Smoyer 2012). Some researchers have explained the social context for HIV risk through social disorganization theory, which hypothesizes that criminal involvement and incarceration increase the risk for HIV infection to both offenders and members of their social network by damaging relationships, and reducing housing and economic stability (Blankenship & Smoyer 2012). Other researchers have used the proximate determinants framework to explain the relationship between an individual’s social context and subsequent HIV risk, reviewing pathways such as injection drug use (IDU), commercial sex work, lower socioeconomic status (SES), mental illness, and sexual partnerships (Boerma & Weir 2005; Epperson et al. 2010a; Khan et al. 2008a). Little research has been done to identify the specific mechanisms of social instability that lead to increased HIV risk in offenders and their partners (Blankenship & Smoyer 2013).

Criminal justice offenders experience numerous personal, social, and economic risk factors that increase their chances of HIV infection. One risk that is particularly evident among those involved in the criminal justice system is drug and alcohol abuse. Nearly two thirds of those under community supervision are drug involved (Taxman et al. 2013). Substance use is associated with increased criminal activity, poorer social networks, and increased sexual risky behaviors when each are evaluated independently (Valera et al. 2009; Pearson et al. 2008; Meyer et al. 2011; Best et al. 2003; Skeem et al. 2009; Staton-Tindall et al. 2007). Therefore, it is necessary to evaluate how substance use influences the relationship between criminal justice risk (risk) and sexual decision-making (Knittel et al. 2013).

Previous research has identified a directional relationship between criminal justice risk factors and sexual risky behaviors. Epperson, El-Bassel, Gilbert, Orellana, and Chang (Epperson et al. 2008) found an association between recent arrest and sexual risk behaviors, including unprotected sex and sex trading. In a subsequent study, Epperson, El-Bassel, Chang, and Gilbert (Epperson et al. 2010b) identified a temporal relationship between criminal justice risk and sexual risk behaviors amongst a group of men with a history of heavy drug use, where criminal justice involvement and short-term incarceration *preceded* an increase in sexual risk behaviors. However, it is unclear whether sexual risk behaviors are directly related to the act of arrest and incarceration or the consequences of larger involvement with the justice system such as poorer social supports, and substance abuse or mental health problems as indicated by theory of social disorganization.

Khan, Epperson, and Comfort (Khan, Epperson et al. 2012) developed the Criminal Justice and HIV Risk Model to describe the relationship between arrest and incarceration on HIV transmission. Khan and colleagues proposed that criminal justice involvement is an underlying determinant that feeds into intermediate pathways which ultimately influences HIV infection. The intermediate determinants as suggested by Khan and colleagues include: 1) disruption of existing sexual networks, 2) reduction of social support which leads to adverse mental health outcomes, 3) establishment of new high risk networks, 4) abandonment of norms, 5) reduction in employment opportunities and financial stability, and 6) increase in alcohol and drug use and abuse. This model of intermediate determinants suggests there are multiple personal and social mechanisms by which criminality increases HIV risk and transmission. This model provides an avenue through which researchers can evaluate specific pathways between criminality and HIV risk and develop interventions that can target modifiable behaviors.

Social networks play an important role in criminal engagement, substance use, and sexual risky behaviors. For instance, improving social networks in adolescence (as a protective factor) and in adult offenders can reduce crime rates and risky behaviors (Cullen 1994; Cullen et al. 1999). Several studies have investigated the connection between social networks and criminal risk, primarily focusing on substance abuse (Best et al. 2003; Skeem et al. 2009; Staton-Tindall et al. 2007; Andrews et al. 2006; Wilson et al. 2012). For instance, spending greater amounts of time with substance abusers has been associated with a greater risk of criminal engagement (Best et al. 2003). Probationers with substance abuse and mental health problems have also been found to have smaller social networks (Skeem et al. 2009). Social support *availability* may be an important indicator of criminality and engagement in risky behaviors. Skeem, Eno Louden, Manchak, Vidal, and Haddad (Skeem et al. 2009) identified the importance of relationship *quality* in which more satisfying personal relationships lead to a reduction in criminal risk.

Additionally, researchers have examined the relationship between social networks during adolescence and their influence on future involvement in the justice system. Cusick, Havlicek, and Courtney (Cusick et al. 2012) elucidate some important findings regarding early social networks and criminal risk later in life. Contrary to previous beliefs regarding the protectiveness of social networks, they found a small association between subjective social support and increased arrest risk. However, they provide two possible explanations: 1) a person’s social network may not be prosocial (e.g., gang involvement may lead to arrest) and 2) even positive, prosocial relationships may not be powerful enough to counterbalance other risk factors faced by vulnerable populations. These findings require further research to determine the mechanisms of prosocial and antisocial social networks and their relationship to risky behaviors. Notably, many studies evaluating social networks focus on *antisocial* (e.g., gang involvement, criminally involved substance users) components and thus, there remains a gap in the literature regarding the effect of *prosocial* components in criminality and health decision-making.

Much of the research conducted on social networks and sexual risky behaviors has addressed the impact of incarceration and relationship disruption with sexual partners. There is a considerable body of literature focusing on the effects of criminal justice involvement on sexual networks, in which incarceration leads to intimate partnership disruption, multiple and concurrent partners, and introduction to high-risk networks and new partners from those high risk networks (Khan et al. 2008a; Khan et al. 2008b; Khan et al. 2009; Khan et al. 2011; Epperson et al. 2011; Rogers et al. 2012). However, few studies have evaluated the effect of non-sexual networks (e.g., parents, siblings, and friends) on sexual risky behaviors and HIV infection. Some researchers have evaluated the role of peer and parental influence on sexual risk behaviors during adolescence (Montgomery et al. 2002; Romer et al. 1994).

The current research draws upon the Criminal Justice Involvement and HIV Risk Model developed by Khan, Epperson, and Comfort (Khan, Epperson et al. 2012) to assess the role of social support on risky behaviors. The purpose of this study was to evaluate the role of social network *quality* (prosocial versus antisocial) and *quantity* (low versus high support availability) on unprotected sex, criminal justice risk, and substance use by modeling and testing path directionality and magnitude between these constructs. The three main objectives were to: 1) establish latent constructs indicative of social network quality and quantity, 2) assess the directionality of the relationship between social support, sexual risk behaviors, criminal justice risk, and substance use, and 3) determine the magnitude of the effect of social network quality or quantity on sexual risk behaviors, criminal justice risk, and substance use. We hypothesized that both quality and quantity of an offender’s social network would be significantly related to sexual risk behaviors, criminal risk, and substance use. Further, we hypothesized that the quality of an offender’s social network would be a more important indicator of criminal risk and sexual risk behaviors than the quantity of available social support.

## Methods

### Data source

Data were extracted from an ongoing randomized controlled trial comparing in-person motivational interviewing to a motivational computer intervention (R01 DA029010-01 National Institute on Drug Abuse). Motivational Assessment Program to Initiate Treatment (MAPIT) is a multisite clinical trial intended to motivate probationers to make early changes in substance use, treatment initiation, and other behaviors related to probation success. The study design has been described elsewhere (Taxman et al. 2015).

Data were collected from 330 newly assigned probationers. To be eligible for participation, probationers must have been at least 18 years old, newly sentenced to probation (within 30 days of sentencing), and reported heavy alcohol use or any illicit drug use within the past 90 days. Informed consent was obtained from all individual participants included in the study. At baseline and 6 months, probationers completed an assessment measuring multiple aspects of their history and current status (e.g., demographics, family history, employment, social support, trauma history, criminal risk, HIV risk behaviors, and substance use). This project was approved by Institutional Review Board at participating universities.

### Model constructs

#### Quality of social support (Exogenous latent variable)

A latent construct (an unobserved underlying measure) indicating the quality of social support within an offender’s social network was created using the family and social section of the Addiction Severity Index Lite (ASI-Lite; (McLellan et al. 1980; McLellan et al. 1999)). The family and social section of the ASI-Lite assesses prosocial (e.g., you help each other with problems, you got along together) and antisocial (e.g., you used illegal drugs together) relationship characteristics of the participant’s parental figures, siblings, spouse/significant other, and friends during the past six months. Items are measured on a five point Likert scale from ‘never’ to ‘always’. Participants who were unable to answer questions for a social network group were set to missing. Items were averaged for each relationship type and possible scores range from 1 – 5, with a higher score indicating a more prosocial relationship and a lower score indicating a more antisocial relationship. This allowed us to examine the measured relationship quality scores (manifest variable) effect on the latent construct of overall social support quality.

#### Quantity of social support (Exogenous latent variable)

The latent construct of quantity or perceived availability of social support was made up of four types of support indicated by the Social Support Survey (SSS; (Sherbourne & Stewart 1991)). The SSS is a 19-item survey measuring the perceived availability of functional social support in four areas: emotional/informational support, tangible support, affectionate support, and positive social interaction. The items on the SSS are measured using a five point Likert scale from ‘none of the time’ to ‘all of the time’. A social support index was calculated for each subscale by averaging the survey items in which a higher score indicated a higher perceived availability of social support and a lower score indicated a lower perceived availability of social support in each area. The separate support quantity indices allowed us to examine the measured social support availability scores effect on the latent construct of overall relationship quantity. The SSS demonstrates acceptable reliability and internal consistency with each subscale exceeding α = 0.50 (Sherbourne & Stewart 1991). The survey has also demonstrated convergent and discriminant validity when compared to other measures of loneliness and family functioning.

#### Sexual risk behavior (Endogenous manifest variable)

Sexual risk behaviors were assessed by evaluating the number of unprotected sex events within the past 30 days reported on the Sexual Risk Behavior Questionnaire (Fisher et al. 2006). The SRBQ is a 110-item self-report measure of HIV/STI risk behaviors within the past 30 days. The frequency of sexual encounters was summed across instances of vaginal sex, anal insertive sex, and anal receptive sex. Data regarding the frequency of condom use was then subtracted from the total number of sexual events to obtain the total number of unprotected, at-risk sexual encounters.

#### Criminal justice risk (Endogenous manifest variable)

Criminal risk was defined as a summary score of the probationer’s prior justice involvement (Taxman et al. 2007). The survey assesses a range of prior justice involvement items that have been found to predict future risk including the number of juvenile and adult arrests, convictions, offenses, incarcerations, and community supervision violations. Possible scores ranged from 0 – 9 where higher scores indicated greater criminal risk. For example, for probationers with 1 – 2 incarcerations equaled one risk point, and those with more than three incarcerations equaled 2 risk points.

#### Substance use (Endogenous manifest variable)

Substance use consisted of daily use for the 90 days prior to the baseline assessment as measured by the Timeline Followback (TLFB; (Sobell & Sobell 1996)). The TLFB measures the frequency of use of nine substances in the 90 days prior to the baseline assessment (i.e., alcohol, marijuana, opiates, cocaine, hallucinogens, barbiturates, inhalants, amphetamines, and prescription pain pills). Alcohol use was measured in daily standard drinks (i.e., 12 oz beer, 5 oz glass of wine, and 1.5 oz of 80 proof liquor), while illicit drug use is measured as frequency of daily use. Substances were collapsed into a single variable to eliminate null values (i.e., participants only had to report using one of the above to meet inclusion criteria) and to account for considerable heterogeneity between substance classes. The reliability and validity of the TLFB is acceptable for assessing self-reported substance use (Fals-Stewart et al. 2000; Sobell et al. 1996). The TLFB demonstrates acceptable convergent and discriminant validity compared to other substance abuse measures (Fals-Stewart et al. 2000).

### Analysis plan

We used a cross-sectional predictive model including structural equation modeling (SEM) to evaluate the direction and association between the variables of interest. Specifically, we examined the relationship between social network and sexual risky behavior, criminal risk, and substance use in offenders. SEM was used to assess the association between several measured variables constructing the latent factors of social network quality and quantity. We examined the composition of the proposed latent variables (social network quality and quantity) by testing the strength of the measurement model, which assessed the factor loadings of the latent variables on the measured variables. Once a satisfactory measurement model was established, we moved to examine the structural model to test the direct paths from the proposed latent factors to the risk behavior measured variables. Adjustments for structural model re-specification were guided by statistical considerations, previous literature, and theory. The analysis controlled for probationer characteristics, such as age, gender, and race. SEM model fit was evaluated with the use of goodness of fit indices (e.g., adjusted goodness of fit index [AGFI], normed fit index [NFI], and root mean square error of approximation [RMSEA]) (Arbuckle 2013; Arbuckle 2006). All analyses were conducted in SPSS Version 20.0 (IBM Corp 2011) and SPSS AMOS Version 22 (Arbuckle 2013).

## Results

### Sample characteristics

This sample included a total of 330 probationers in Dallas, TX (*N* = 188) and Baltimore, MD (*N* = 142). Sixty-five percent of participants were Black, 22 % were White, and 13 % were another race, see Table 1. Participant ages ranged from 18 to 62 years (*M* = 35.15, *SD* = 11.77). Most participants were male (68 %). Twenty-one percent of participants were considered to be low risk, 44 % were of moderate risk, and 35 % were of high criminal risk. In terms of sexual orientation, 88 % were heterosexual, 5.5 % gay/lesbian, and 5.6 % bisexual. This sample mainly consisted of single/never-married probationers (65.8 %), while 20.3 % reported being divorced, widowed, or separated, and 13.6 % were currently married. The majority of participants reported having sex at least once within the past 30 days (30.6 % reported abstinence). On average, participants reported 11.2 sexual encounters. The average number of unprotected sexual encounters in the past 30 days was 8.6. Of the participants who reported having sex in the past 30 days, 46.7 % (*N* = 154) reported not using a condom.

On average, probationers reported relationship quality scores ranging from 3.7 to 3.9 on a scale of 1 – 5. This approximate range corresponds to relationships being reported as positive or prosocial from ‘sometimes’ to ‘often’. On average, probationers also reported a higher availability of different kinds of social support should they need it (i.e., emotional, tangible, affectionate, and positive social interaction) with mean scores ranging from 3.6 to 3.9. This is characterized by social support being available ‘some of the time’ to ‘most of the time’.

### Measurement model testing

Results from separate confirmatory factor analyses and standardized factor loadings for the two latent variables, social support quality and social support quantity are presented in Table 2. Factor variances were free to vary, while one random indicator regression weight was constrained to 1.0 per latent construct. All measured indicators were significant. The measurement model for social support quality latent construct was good: CMIN/df = 1.50 (*p =* .223*,* df = 2), AGFI = .978, NFI = .969, RMSEA = .038, Hoelter’s *N* = 685. The measurement model for social support quantity latent construct was acceptable: CMIN/df = 2.41 (*p =* .090*,* df = 2), AGFI = .964, NFI = .996, RMSEA = .064, Hoelter’s *N* = 426.

**Table 1:** Participant characteristics

	Mean or frequency	SD or percentage
Sociodemographics		
Age	35.15	11.77
Race		
Black/African American	*N* = 214	64.8 %
Caucasian	*N* = 74	22.4 %
Other	*N* = 42	12.7 %
Gender		
Male	*N* = 224	67.9 %
Female	*N* = 104	31.5 %
Transgender (Male to Female)	*N* = 2	0.6 %
Sexual Orientation		
Heterosexual	*N* = 291	88.2 %
Gay/Lesbian	*N* = 18	5.5 %
Bisexual	*N* = 19	5.8 %
Other	*N* = 2	0.3 %
Relationship Status		
Never Married	*N* = 217	65.8 %
Married	*N* = 45	13.6 %
Divorced/Widowed/Separated	*N* = 67	20.3 %
Criminal Risk Level		
Low	*N* = 70	21.2 %
Medium	*N* = 145	43.9 %
High	*N* = 115	34.8 %
Sexual Events (Previous 30 Days)	11.24	26.98
Unprotected Sexual Events	8.60	15.03
Social Support Quality		
Parents	3.83	0.58
Siblings	3.91	0.57
Friends	3.78	0.76
Spouse/Significant Other	3.70	0.58
Social Support Quantity		
Emotional Support Availability	3.64	1.08
Tangible Support Availability	3.66	1.27
Affectionate Support Availability	3.90	1.25
Positive Social Interaction Availability	3.83	1.17

**Table 2:** Measurement model standardized loadings

Latent factor	Measured indicator	Loading	*R* ^*2*^
Social Support Quality	Parents	1.00	.28
	Siblings	1.14	.40
	Spouse/Significant Other	0.54	.09
	Friends	1.08	.19
Social Support Quantity	Emotional/Informational Support	1.00	.68
	Tangible Support	1.26	.78
	Affectionate Support	1.27	.82
	Positive Social Interaction	1.19	.82

### SEM model fit

The chi-squared goodness of fit test indicated the specified model was significantly different from the saturated model (*χ* ^2^ = 133.05, df = 67, *p* < .001). However, the discrepancy function may be overly sensitive to small differences in fit between the model and the data. The ratio of chi-square to degrees of freedom was 1.99. It has been proposed that a ratio below 2.0 indicates a good fit. The AGFI indicated the proportion of the observed covariance explained by the model covariance was 91.3 % after adjusting for model complexity. The proportion of improvement of the overall model fit relative to the independence model was good (NFI = 0.912), indicating the proposed model was 91.2 % better than the independence model. The difference in the observed and model-implied covariances was acceptable (RMSEA = 0.055).

### Structural model testing

We initially tested a mediated model of social support quality and quantity between criminal risk and sexual risk behaviors. Direct paths were measured from the exogenous manifest variable, criminal risk, to the exogenous latent constructs, social support quality and quantity. Then direct paths were measured from the exogenous latent factors to sexual risk behaviors, an endogenous manifest variable. However, this model did not have adequate fit (data not shown).

**Fig. 1:**
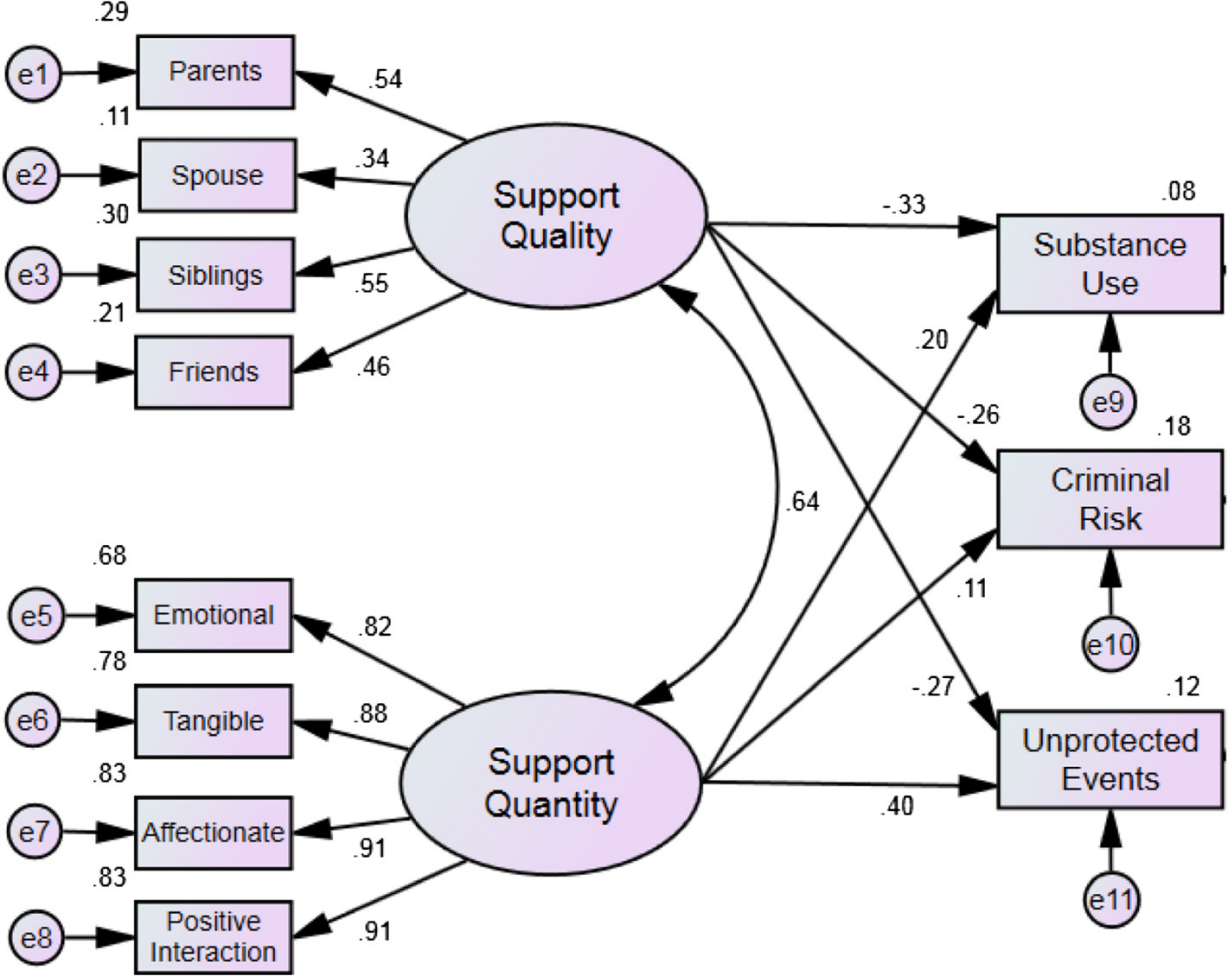
Structural Model of Social Support Quality and Quantity on Risk Taking Behaviors. Final structural model of social network quality and quantity and its relationship to risk taking behaviors. Relationships between dimensions of social support and risk taking behaviors are represented by standardized parameter estimates

We treated each manifest variable (criminal risk, sexual risk behaviors, and substance use) as an outcome (endogenous) of social support quality and quantity as factors of social support can affect criminal activity, substance use, and sexual risk behaviors from an early age. Direct paths from social support quality were significantly negatively associated with sexual risk behaviors (*p* < .05), criminal risk (*p* < .05), and substance use (*p* < .05), see Fig. 1. Probationers with a higher quality of social support had reduced sexual risk behaviors in the past 30 days, fewer days of substance use in the past 90 days, and a lower level of criminal risk. Direct paths from social support quantity had significant positive associations with sexual risk behavior (*p* < .05) and substance use (*p* < .05). Probationers with a higher availability of social support had more unprotected sexual encounters in the past 30 days and more days of substance use in the past 90 days. Social support quantity was not significantly associated with criminal risk. There was significant positive covariance between the latent constructs of social support quality and social support quantity; as the quantity or availability of support increased so did the quality of social support. Each endogenous variable, criminal risk, substance use, and sexual risk behaviors, were controlled for participant’s age, gender, and race (data not shown). Additionally, we evaluated this model while controlling for marital status and sexual orientation. The directionality and significance of our reported findings were unaffected, however, the model fit was reduced so we chose to present the more parsimonious model.

## Discussion

This paper evaluated the relationship between social support quality (prosocial versus antisocial) and quantity (low versus high) of an offender’s social network on criminal risk, sexual risk behaviors, and substance use. We evaluated the directionality and magnitude of these relationships by modeling and testing paths through SEM. Two proposed unobserved latent constructs reflecting a probationer’s current social support quality and quantity were created to perform the SEM. The measured indicators were significantly related to our proposed latent constructs and each indicated a good model fit. Although this model met the recommended guidelines for acceptability, there is still room for improvement and variance unexplained by the proposed model.

The SEM model indicated the quality of social support in an offender’s immediate social network was significantly negatively associated with sexual risk behaviors, criminal risk, and substance use. As the quality of one’s social support increases (e.g., helping each other with problems, enjoying the time spent together, not fighting), sexual risk behaviors, criminal risk, and substance use decrease. Our findings regarding support network quality are similar to previous literature that found a reduction in criminal risk based on relationship quality (Skeem et al. 2009). For instance, Cusick, Havlicek, and Courtney (Cusick et al. 2012) highlight the importance of early social networks in predicting later criminal risk. They found that subjective social networks were associated with increased arrest risk; however, they did not assess the quality of these networks. The significance of prosocial support on criminality and health decision-making is evident. Other researchers have found that disruptions in sexual networks can lead to increases in sexual risky behaviors (Khan et al. 2009; Khan et al. 2011; Rogers et al. 2012). Our findings help expand this relationship by evaluating the effect of non-sexual networks (e.g., parents, siblings, and friends) on sexual risky behaviors and HIV infection in a criminal justice population.

Our proposed model determined that the quantity of social support was significantly positively associated with sexual risk behavior and substance use. Therefore, as the amount of social connections increase, sexual risk behavior and substance use increase. However, the quantity of an offender’s social support was not significantly associated with future criminal risk. This finding may be due in part to the limited understanding of whether the amount of perceived social support would be enough to counteract risk factors associated with sexual risk behavior and substance use in this population. Further research is needed to determine the relationship between social support quantity and risky sexual behavior and substance use.

We found a paradoxical relationship between social network quality and quantity in relation to engagement in risky behaviors. This model indicated that social support quality served as a protective factor while social support availability served as a risk factor for engaging in risky behaviors. The proposed model provided some evidence supporting our hypothesis that the quality of an offender’s social network can act as a protective factor against sexual risk behaviors, criminal risk, and substance use. However, it is interesting to note that we found a reverse relationship in which social support availability led to increases in sexual risk behaviors and substance use. There are several possible reasons for this paradoxical relationship. First, the observed associations may represent a situation in which individuals engaging in risky sexual behavior and substance use are members of a larger social network that proliferates risk taking behaviors. The question may not be the quantity of social relationships that matters, but whether or not they are part of a social network/community where this kind of risky/criminal behavior is normative. Second, social support availability may function differently depending on the nature of the population. This model indicated that quality and quantity were positively associated with each other. At first glance, this positive relationship seems paradoxical given that these two latent constructs were inversely related to engagement in risky behaviors. However, our results suggest that the two constructs function differently. Presumably one cannot have social support quality without at least some quantity, but the reverse may not necessarily be true. People can certainly have a large social network that is of poor (or good) quality. The sheer presence of social support and/or a larger social network, on average, increases a person’s opportunities for sexual risky behaviors and substance use if the family and peer network also engage in these behaviors. However, a person will not be able to achieve a high quality social support network without also having at least some quantity. Third, it is possible that all probationers indeed have some quantity and there is limited variability in this construct. If so, quality would serve as a driving factor for reduced engagement in risky behaviors. Finally, it is also possible that probationers may have over-estimated their social support quality or availability when responding to survey questions in order to answer in the socially appropriate direction or negate embarrassment from the interviewer. It will be important for future research to investigate these hypotheses in more detail.

Here we found two constructs that were positively related to each other, but one had a protective factor on the outcome, while the other one acted as a risk factor. We propose two analogies to explain this relationship. First, it is possible for calorie intake and exercise to be positively correlated, and at the same time have inverse relationships on weight. Presumably those who exercise more also consume more calories, even though calorie intake has a positive association with weight and exercise has a negative association with weight. Our findings may also be equated to purchasing a diamond. A small diamond of pure quality can cost the same as a large diamond of poor quality; the importance of quality can trump the quantity. The implication here is to focus on developing interventions that change/replace low quality contacts with more supportive/high quality contacts.

It is generally recognized that individuals who are involved in the criminal justice system are at a greater risk for HIV due to an increased number of risk factors and engagement in risky behaviors (Maruschak & Beavers 2010; Center for Disease Control and Prevention 2012). Therefore, it is important for public health practitioners to develop interventions tailored to this vulnerable population. Given the directionality and magnitude of the relationships identified in this SEM, sexual health interventions should target at-risk youth through interventions that change the nature of social networks, which can result in later reductions of criminal activity, substance use, and HIV and STD risk. Previous research has shown that social networks may serve as a protective factor against criminal justice involvement and risky behaviors such as sexual activity and substance use (Cullen 1994; Cullen et al. 1999). Additionally, social support skills enhancement may be beneficial for reducing sexual risky behaviors (El-Bassel et al. 1995; El-Bassel et al. 1997). Given the previously identified risk between arrest and HIV infection (Khan et al. 2012a; Khan et al. 2012b), interventions focusing on condom use and partnership reduction should be targeted towards adolescents prior to their potential involvement in the criminal justice system. Preventative measures given at the right time in the causal pathway may indeed disrupt the chain between poor quality social networks, criminal risk, substance use, and sexual risky behaviors.

### Limitations

There are limitations to this research that should be considered when interpreting these results. First, this is a cross-sectional study design in which temporality cannot be determined when evaluating these relationships; we can only make statements about the strength of the associations. Second, this SEM met the minimum guidelines for goodness of fit, but there is still room for improvement and much of the variance is still unexplained. More research is necessary to elucidate the relationships between risky behavior and non-sexual network social support. Third, the Sexual Risk Behavior Questionnaire utilized in this study did not assess for unprotected oral sex, which is also a risk factor for STD/HIV transmission. Fourth, due to modeling constraints we were unable to evaluate interaction effects of social support quality and quantity, further research is needed. Additionally, subgroup analyses were not conducted because of a relatively small sample size and restricted range of responses. Finally, due to limited frequency of occurrence and/or considerable variability of different sexual risk behaviors (e.g., multiple concurrent partnerships, history of STD/HIV, and sex under the influence of substances) we only used the number of unprotected sex events to determine HIV risk. However, this was deemed the best overall indicator of STD/HIV risk because the frequency of unprotected events can place the individual at higher/lower risk for STDs.

## Conclusions

This study used SEM to evaluate the role of probationer social support quality and quantity on risky behaviors. We found that better quality social support was negatively associated with substance use, criminality, and engagement in sexual risk behaviors. The quantity or availability of social support was positively associated with substance use and engagement in sexual risky behaviors. Our results provide a model with which to evaluate these associations further to determine how the criminal justice system might address the high rates of substance use and HIV or STDs among offenders. Improvements in the quality of social support systems for probationers and a minimization of contact with poor quality networks can have a significant impact on engagement in risky behaviors, which pose significant public health costs to the United States.

## References

[CR1] Andrews DA, Bonta J, The Psychology of Criminal Conduct (2006). Cincinnati.

[CR2] Arbuckle JL (2006). AMOS 7.0 User’s Guide.

[CR3] Arbuckle JL (2013). SPSS Amos.

[CR4] Best D (2003). Getting by with a little help from your friends: the impact of peer networks on criminality in a cohort of treatment-seeking drug users. Addictive Behaviors.

[CR5] Blankenship KM, Smoyer AB, Sanders B, Thomas TF, Deeds BG (2012). Between spaces: Understanding movement to and from prison as an HIV risk factor. Crime, HIV and Health: Intersections of Criminal Justice and Public Health Concerns.

[CR6] Blankenship KM, Smoyer AB, Sanders B, Thomas TF, Deeds BG (2013). Between spaces: Understanding movement to and from prison as an HIV risk factor. Crime, HIV and health: intersections of criminal justice.

[CR7] Boerma JT, Weir SS (2005). Integrating demographic and epidemiological approaches to research on HIV/AIDS: the proximate-determinants framework. Journal of Infectious Diseases.

[CR8] Center for Disease Control and Prevention (2012). HIV in correctional settings.

[CR9] Cullen, F.T. (1994). Social support as an organizing concept for criminology: Presidential address to the academy of criminal justice sciences*. Justice Quarterly*, *11*(4), 527-559.

[CR10] Cullen FT, Wright JP, Chamlin MB (1999). Social support and social reform: A progressive crime control agenda. Crime and Delinquency.

[CR11] Cusick GR, Havlicek JR, Courtney ME (2012). Risk for arrest: the role of social bonds in protecting foster youth making the transition to adulthood. American Journal of Orthopsychiatry.

[CR12] El-Bassel N (1995). Preventing HIV/AIDS in drug-abusing incarcerated women through skills building and social support enhancement: preliminary outcomes. Social Work Research.

[CR13] El-Bassel N (1997). Skills building and social support enhancement to reduce HIV risk among women in jail. Criminal Justice and Behavior.

[CR14] Epperson MW (2008). Increased HIV risk associated with criminal justice involvement among men on methadone. AIDS and Behavior.

[CR15] Epperson MW (2010). Assessing criminal justice involvement as an indicator of human immunodeficiency virus risk among women in methadone treatment. Journal of Substance Abuse Treatment.

[CR16] Epperson MW (2010). Examining the temporal relationship between criminal justice involvement and sexual risk behaviors among drug-involved men. Journal of Urban Health.

[CR17] Epperson MW (2011). A longitudinal study of incarceration and HIV risk among methadone maintained men and their primary female partners. AIDS and Behavior.

[CR18] Fals-Stewart W (2000). The timeline followback reports of psychoactive substance use by drug-abusing patients: psychometric properties. Journal of Consulting and Clinical Psychology.

[CR19] Fisher JD (2006). Clinician-delivered intervention during routine clinical care reduces unprotected sexual behavior among HIV-infected patients. Journal of Acquired Immune Deficiency Syndromes.

[CR20] Herberman EJ, Bonczar TP (2014). Probation and parole in the United States, 2013.

[CR21] IBM Corp (2011). IBM SPSS Statistics for Windows, Version 20.0.

[CR22] Khan, M. R., Epperson, M., Comfort, M. (2012). *The criminal justice involvement and HIV risk model: A novel conceptual model that describes the influence of arrest and incarceration on STI/HIV transmission*, in *American Public Health Association Annual Meeting*. San Francisco, CA: American Public Health Association.

[CR23] Khan MR (2008). Incarceration and risky sexual partnerships in a southern US city. Journal of Urban Health.

[CR24] Khan MR (2008). Timing and duration of incarceration and high-risk sexual partnerships among African Americans in North Carolina. Annals of Epidemiology.

[CR25] Khan MR (2009). Incarceration and high-risk sex partnerships among men in the United States. Journal of Urban Health.

[CR26] Khan MR (2011). Dissolution of primary intimate relationships during incarceration and implications for post-release HIV transmission. Journal of Urban Health.

[CR27] Khan MR (2012). Longitudinal associations between adolescent alcohol use and adulthood sexual risk behavior and sexually transmitted infection in the United States: assessment of differences by race. American Journal of Public Health.

[CR28] Khan MR (2012). Adolescent criminal justive involvement and adulthood sexualy transmitted infection in a national representative US sample. Journal of Urban Health: Bulletin of the New York Academy of Medicine.

[CR29] Knittel AK (2013). Incarceration and sexual risk: examining the relationship between men’s involvement in the criminal justice system and risky sexual behavior. AIDS and Behavior.

[CR30] Maruschak LM, Beavers R (2010). HIV in prisons, 2007–08.

[CR31] McLellan AT (1980). An improved diagnostic evaluation instrument for substance abuse patients. The Addiction Severity Index. The Journal of Nervous and Mental Disease.

[CR32] McLellan AT (1999). Addiction Severity Lite - CF.

[CR33] Meyer JP, Springer SA, Altice FL (2011). Substance abuse, violence, and HIV in women: a literature review of the syndemic. Journal of Womens Health (Larchmt).

[CR34] Montgomery SB (2002). Gender differences in HIV risk behaviors among young injectors and their social network members. The American Journal of Drug and Alcohol Abuse.

[CR35] Pearson FS (2008). Substance use, mental health problems, and behavior at risk for HIV: evidence from CJDATS. Journal of Psychoactive Drugs.

[CR36] Rogers SM (2012). Incarceration, high-risk sexual partnerships and sexually transmitted infections in an urban population. Sexually Transmitted Infections.

[CR37] Romer D (1994). Social influences on the sexual behavior of youth at risk for HIV exposure. American Journal of Public Health.

[CR38] Sherbourne CD, Stewart AL (1991). The MOS social support survey. Social Science and Medicine.

[CR39] Skeem J (2009). Social networks and social control of probationers with co-occurring mental and substance abuse problems. Law and Human Behavior.

[CR40] Sobell LC, Sobell MB (1996). Timeline Followback user’s guide: A calendar method for assessing alcohol and drug use.

[CR41] Sobell LC (1996). The reliability of the Alcohol Timeline Followback when administered by telephone and by computer. Drug and Alcohol Dependence.

[CR42] Staton-Tindall M, Royse D, Leukfeld C (2007). Substance use criminality, and social support: an exploratory analysis with incarcerated women. American Journal of Drug and Alcohol Abuse.

[CR43] Taxman FS, Perdoni ML, Caudy M (2013). The plight of providing appropriate substance abuse treatment services to offenders: Modeling the gaps in service delivery. Victims and Offenders.

[CR44] Taxman FS (2007). Screening, assessment, an referral practices in adult correctional settings: A national perspective. Criminal Justice Behavior.

[CR45] Taxman, F.S., Walters, S. T., Sloas, L. B., Lerch, J., & Rodriguez, M. (2015). Motivational tools to improve probationer treatment outcomes. *Contemporary Clinical Trials), 43*, 120-128. 10.1016/j.cct.2015.05.016 PMC4522391 26009023

[CR46] Valera P (2009). Substance use and HIV-risk behaviors among young men involved in the criminal justice system. The American Journal of Drug and Alcohol Abuse.

[CR47] Wilson ME, Taxman FS, O’Grady KE (2012). The relationship of social networks to HIV risk behaviors from a sample of probationers in a randomized trial. The Prison Journal.

